# High prevalence of multidrug-resistant *Salmonella enterica* in Thailand food markets: insights from complete genome and phenotypic characterization of ESBL-producing strains

**DOI:** 10.1128/spectrum.02129-25

**Published:** 2025-10-31

**Authors:** Phutthaphorn Phaophu, Nalumon Thadtapong, Samantha E. Wirth, Anna S. Gray, Sunisa Dangsuk, Natharin Ngamwongsatit, Ratchaneewan Aunpad, Soraya Chaturongakul

**Affiliations:** 1 Center for Advanced Therapeutics, Institute of Molecular Biosciences, Mahidol University 98841, Nakhon Pathom, Thailand; 2 Department of Medical Sciences, National Institute of Health, Ministry of Public Health 37694 , Nonthaburi, Thailand; 3 New York State Department of Health, Wadsworth Center 116287, Albany, New York, USA; 4 Department of Clinical Sciences and Public Health, Faculty of Veterinary Science, Mahidol University 71652, Nakhon Pathom, Thailand; 5 Graduate Program in Biomedical Sciences, Faculty of Allied Health Sciences, Thammasat University 37698, Pathum Thani, Thailand; 6 Pornchai Matangkasombut Center for Microbial Genomics (CENMIG), Faculty of Science, Mahidol University 98842, Bangkok, Thailand; University of Maryland Eastern Shore, Princess Anne, Maryland, USA

**Keywords:** *Salmonella* spp., food safety, whole genome sequencing, complete genome, multidrug-resistant (MDR), extended-spectrum β-lactamase (ESBL)

## Abstract

*Salmonella* contamination in the food chain is a common root of foodborne outbreaks. The transmission of antimicrobial resistance among *Salmonella* and food bacteria raises global public health concern. As a major food exporter and travel hub in Southeast Asia, Thailand is particularly susceptible. To better understand the circulating *Salmonella*, we characterized 74 non-typhoidal *Salmonella* isolates previously obtained from Thailand food markets. Whole genome sequencing, drug susceptibility testing, and host invasion efficiency assays were performed. The most prevalent serotype was *Salmonella* serotype Rissen (32.43%) followed by Derby (12.16%). Fifty-two isolates were multidrug-resistant (MDR), with their primary resistance pattern being ampicillin, streptomycin, and tetracycline resistance. The most resistant strain was *S*. Rissen exhibiting resistance to 13 tested drugs, including third-generation cephalosporin. Six cephalosporin-resistant isolates were identified, four of which were extended-spectrum β-lactamase (ESBL)-producing strains harboring the *bla*_CTX-M-14_ or *bla*_CTX-M-55_ ESBL gene. Complete genomes showed that three ESBL-producing isolates contained plasmid-mediated ESBL, and the other was chromosomal-mediated. *In vitro* invasion efficiencies of ESBL-producing strains into mucous-producing human colorectal adenocarcinoma HT29-MTX-E12 cells were compared to the reference strain *Salmonella* Typhimurium LT2. Statistically significant higher invasion efficiency in the chromosomal-mediated ESBL strain was found. Interestingly, the ESBL plasmid detected during this study was highly similar to a plasmid previously isolated from a Thai patient. This study highlights that the majority of *Salmonella* isolated from Thai foods are MDR and potentially ESBL-producing strains, highlighting the need for continued surveillance and intervention strategies.

**IMPORTANCE:**

In Thailand, fermented foods are typically consumed raw, and pork is considered a delicacy of Thai cuisine. The presence of multidrug resistant (MDR) foodborne pathogens in these food types raises concern and presents a risk to public health. Here, we report that *Salmonella* Rissen was the most prevalent serotype isolated from these food samples in Thailand. All isolates carried virulence gene clusters crucial for pathogenesis, and more than 70% of isolates were MDR strains. Four of the MDR strains were ESBL-producing. Whole genome sequence analysis and phenotypic characterizations revealed that chromosome-mediated ESBL strains possessed higher *in vitro* invasion efficiency than plasmid-mediated ESBL strains. This study highlights two key public health threats: the risk of acquiring difficult-to-treat MDR *Salmonella* infections from undercooked food and the circulation of AMR plasmids in fresh markets in Thailand.

## INTRODUCTION

Non-typhoidal *Salmonella* (NTS) serotypes are highly prevalent and exhibit broader host range (1). In a comprehensive study of 2006 data, Southeast Asia was estimated to have 22.8 million NTS gastroenteritis cases, and 37,600 deaths are reported (2). Multidrug-resistant (MDR) *Salmonella* poses a public health burden with serious outcomes. Increased disease severity, especially in immunocompromised patients, results in prolonged hospitalization and treatment complications or failures leading to death (3, 4). The emergence of extended-spectrum β-lactamase (ESBL)-producing *Salmonella* has led to the increase of resistant strains to penicillin, including third- and fourth-generation cephalosporins. Additionally, ESBL *Salmonella* can transmit their antimicrobial resistance (AMR) genes via horizontal gene transfer within the same food matrix (5). The increased incidence of MDR *Salmonella* is a significant risk to human health and a burden on healthcare systems. In order to help prioritize research and develop strategies to combat AMR, the World Health Organization (WHO) has classified *Salmonella* as a high-priority pathogen (6).

*Salmonella* serotypes Enteritidis and Typhimurium are the most common and widely distributed serotypes reported globally (7). Currently, *S*. Typhimurium is not listed in the top five NTS serotypes associated with human clinical disease in Thailand because monophasic variant serotype (*S*. 4,[5],12:i:-) is becoming more common (8). Even though cases of salmonellosis due to an emerging serotype Rissen are relatively rare compared to *S*. Enteritidis, *S*. Rissen has been reported as the most prevalent serotype isolated from swine and pork products (9, 10). The MDR profiles of *S*. Rissen and persistence in the environment and animals have been documented over the past decade (11).

Our recent report found that *Salmonella* contamination in retail pork meats and fermented foods usually consumed raw was detected in 54% of samples by culture methods (12). However, *Salmonella* serotypes and genes associated with AMR and virulence genes are still unknown. To investigate the prevalence of *Salmonella* serotypes and the occurrence of AMR and virulence genes among these isolates, this study focused on serotype prevalence and detection of MDR strains and ESBL-producing *Salmonella* strains by leveraging genomic analyses. The correlation between genotype (AMR and virulence genes) and phenotype (antibiotic resistance) was investigated. Furthermore, we performed both short-read and long-read sequencing, followed by hybrid assembly on the four ESBL-producing strains in order to report their complete genomes. To determine the invasion efficiency of these ESBL-producing strains compared to *Salmonella* Typhimurium LT2, an invasion assay into mucus-producing cell line HT29-MTX-E12 was performed. In summary, this study has provided valuable details about a subpopulation of food-isolated *Salmonella* in Thailand. Specifically, we describe the serotype distribution, prevalence of AMR patterns and virulence genes, and prevalence of ESBL-producing strains and their ability to invade human intestinal cells.

## RESULTS

### Prevalence of *Salmonella* in food samples

**Fig 1:**
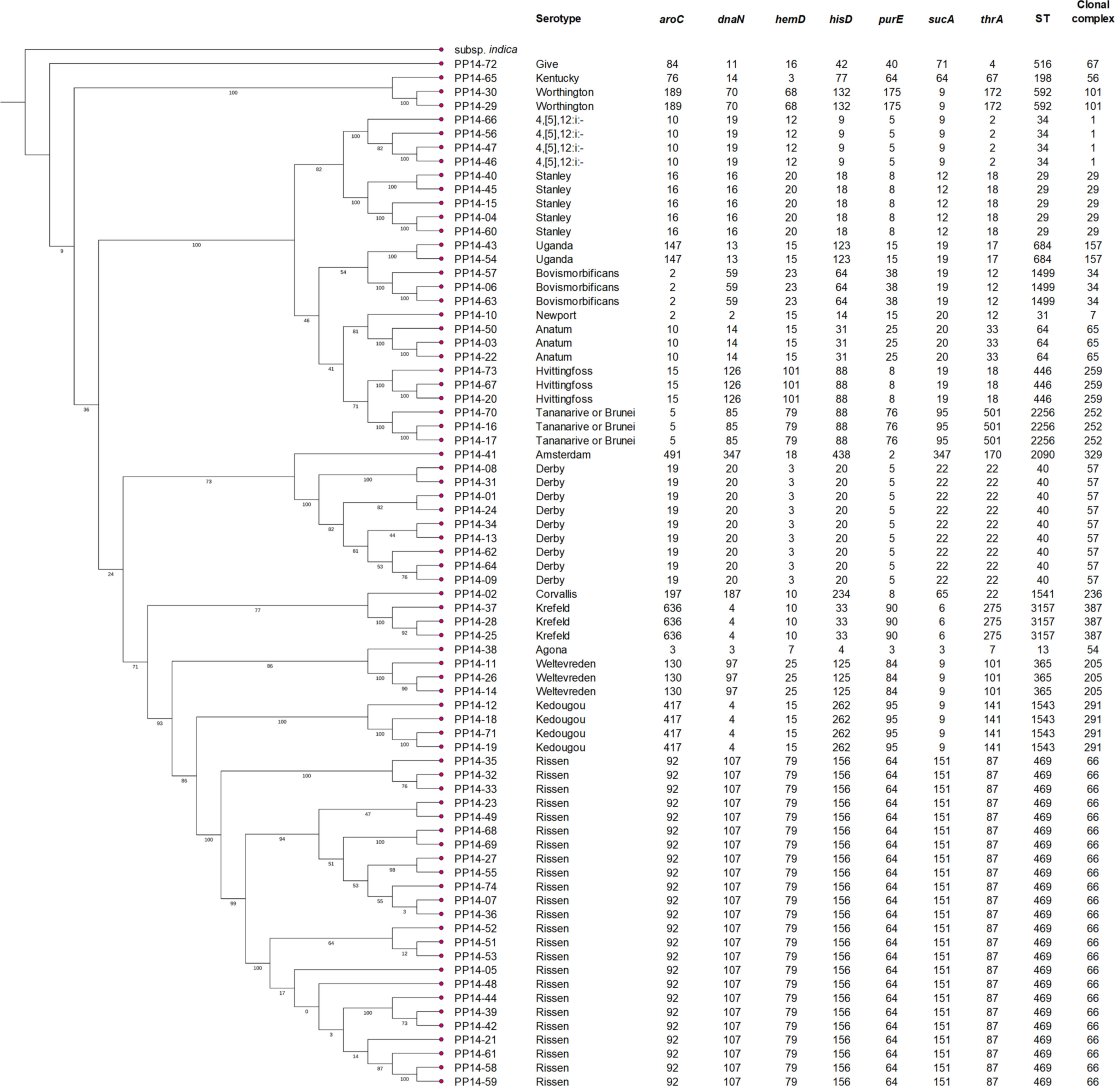
Core genome phylogenetic tree of 74 *Salmonella* isolates with their serotypes, MLSTs, and STs. *Salmonella enterica* subsp. *indica* was used as an outgroup.

Seventy-four *Salmonella* strains were isolated from 100 food samples including raw retail pork meats, fermented pork sausages, fermented fishes, and fermented fish entrails. They were obtained from five different regions in Thailand (12). Of these, 59 strains were isolates from 38 retail pork meat samples, and the other 15 isolates were from 11 fermented food samples. Among the fermented food isolates, 12 were from eight fermented pork sausage samples, and three were from three fermented fish samples (Fig. S1). Whole genome sequencing and analyses of all isolates were performed. The quality of the assembled genome sequences was assessed as presented in Table S1. According to serotyping by SeqSero2 and multilocus sequence typing (MLST) analysis, 19 different serotypes and 19 sequence types (STs) were found (Fig. 1; Table S2) with one ST associated with each serotype. The most prevalent serotypes were serotype Rissen (24/74, 32.43%), followed by Derby (9/74, 12.16%), Stanley (5/74, 6.76%), 4,[5],12:i:- and Kedougou (4/74, 5.41%), and Anatum, Bovismorbificans, Hvittingfoss, Krefeld, Tananarive or Brunei, and Weltevreden (3/74, 4.05%). Moreover, serotype Rissen showed the highest prevalence in both types of food samples, consistent with previous reports on the prevalence of *S*. Rissen especially in the swine production chain and at the market (11, 13).

### Genotypic AMR profiling

**Fig 2:**
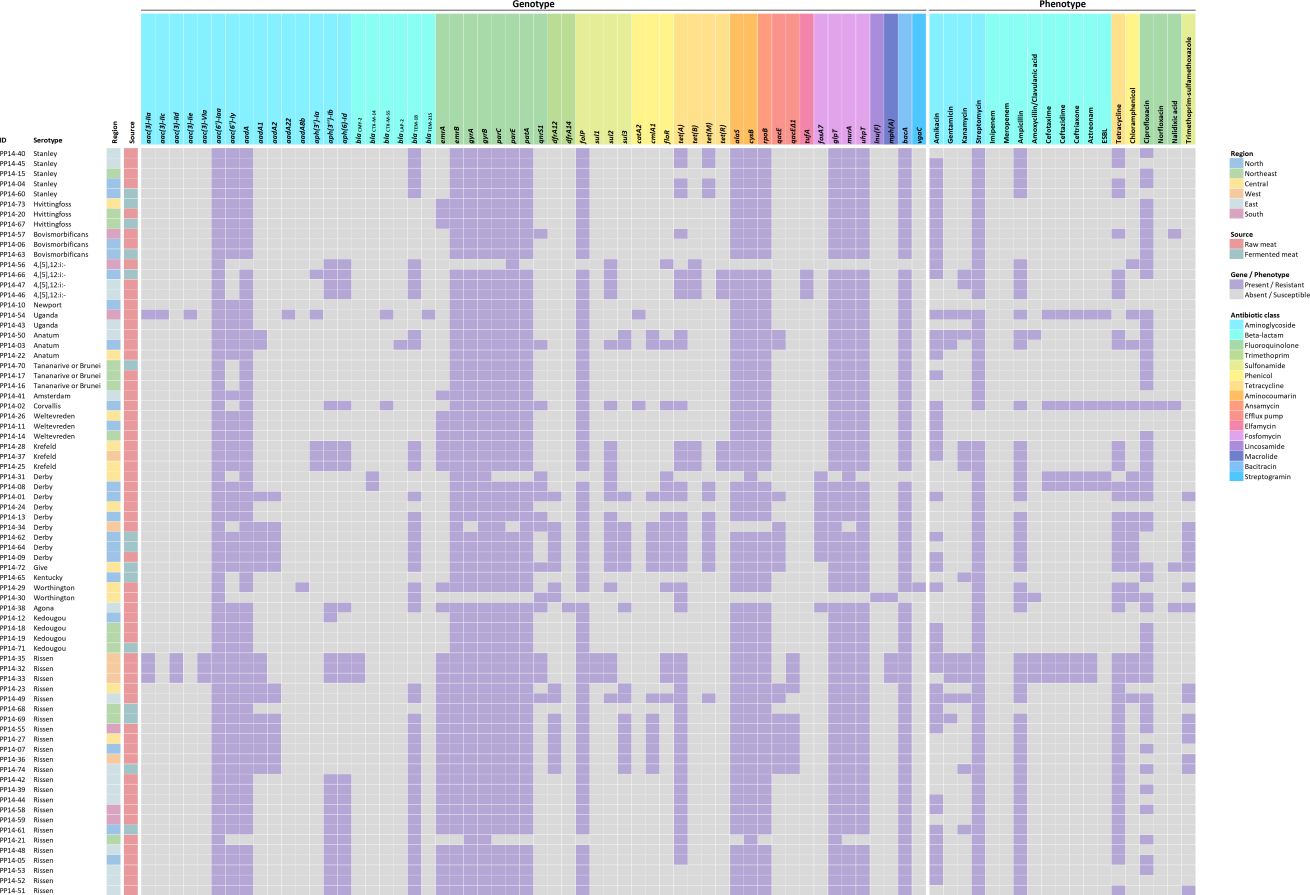
Heatmap of AMR pattern of *Salmonella* isolates based on their genotypic and phenotypic information. Presence and absence are respectively shown in purple and gray. Different colors in AMR genes and antibiotics represent different antibiotic classes which are labeled next to the heatmap.

Based on three AMR databases, a total of 56 AMR genes were identified in this study, as shown in Fig. 2 and Table S3. Detected genes confer resistance to 15 antibiotic classes and one efflux pump. Among 74 isolates, all strains harbor the *aac(6')-Iaa* gene, encoding an aminoglycoside-modifying enzyme. Seventy-two *Salmonella* strains (97.30%) harbor *gyrB* gene encoding DNA gyrase subunit B and conferring resistance to the fluoroquinolone drug class. Furthermore, resistance genes to aminoglycoside class (*aadA*), fluoroquinolone class (*emrB* and *gyrA*), aminocoumarin class (*cysB*), fosfomycin class (*glpT*), and bacitracin class (*bacA*) were frequently found, accounting for 95.95% (71/74 samples). Focusing on β-lactam groups, *bla*_TEM-1_ gene showed the highest frequency (44/74, 59.46%), while few strains contained *bla*_CMY-2_ (3/74, 4.05%), *bla*_CTX-M-14_, or *bla*_CTX-M-55_ (2/74, 2.70%). Sulfonamide resistance gene, *folP*, was found in 70 strains (94.59%). Moreover, *tet(A*) was the most prevalent tetracycline resistance gene discovered in 46 isolates. Fortunately, *mcr* gene, mobilized colistin resistance, was not detected in this *Salmonella* collection.

*In silico* plasmid typing showed that 17 plasmid replicon types were detected (Table S4). Fifty-two isolates harbor at least one plasmid, and most of them carry Col(pHAD28) (36/74, 48.65%). The second most commonly found plasmid type was Col440I (11/74, 14.86%). *S*. Derby strains PP14-08 and PP14-31 had the highest number and variety of plasmids which were Col(pHAD28), IncFIA(HI1), IncHI1A, and IncHI1B(R27). There was no correlation between serotype and detected plasmid replicon. However, 22 strains did not match with known plasmid types in the database.

Mobile genetic element (MGE) detection revealed that MITEEc1, a miniature inverted-repeat transposable element (MITE), was present in all isolates along with 34 MGE types found in this study (Fig. S2, Table S5). Most *S*. Rissen isolates shared genome elements, including insertion sequences (IS) IS*Ecl10*, IS*Kpn2*, and MITEEc1. Half of the isolates also harbored IS*26*, which has been reported to frequently associate with resistance determinants (14). Most of the *S*. Derby isolates shared the same MGE profile consisting of IS*Ecl10*, IS*Kpn26*, IS*26*, cn_5129_IS*Vsa3* (composite transposon), IS*Vsa3*, IS*630*, and MITEEc1. Few serotypes were detected that carried transposons, namely, Stanley, Krefeld, and Rissen.

### Virulence-associated genes and SPIs in *Salmonella* isolates

**Fig 3:**
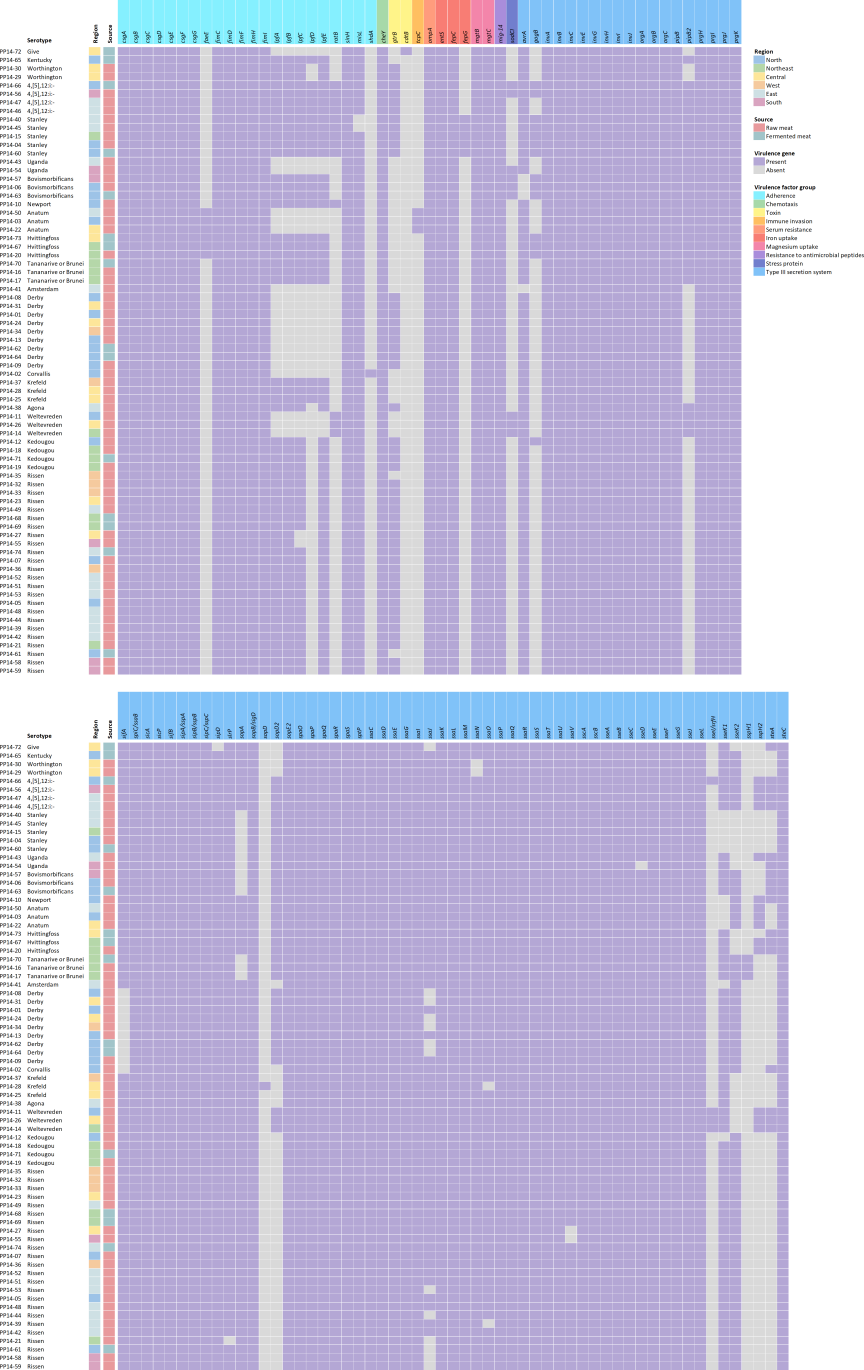
Heatmap of virulence genes detected in *Salmonella* isolates. Presence and absence are respectively shown in purple and gray. Different colors in virulence genes represent different virulence factor groups which are labeled next to the heatmap.

Based on the Virulence Factor Database (VFDB) database, a total of 110 virulence genes were detected. Their properties were predicted (Table S6), and virulence gene patterns are shown in Fig. 3. All studied strains harbored virulence genes *csgABCDEF* (*csg* cluster, adherence), *fimCDFHI* (fimbriae operon, adherence), *cheY* (chemotaxis), *ompA* (outer membrane protein, invasion), *entS* (enterobactin, iron uptake), *fepC* (ferric enterobactin uptake, iron uptake), *mgtBC* (magnesium transporter, magnesium uptake), *mig-14* (resistant to antimicrobial peptides), and genes involved in type III secretion system (T3SS) such as of *invABCEGHIJ* (*inv* operon), *prgHIJK-orgABC* operon, *sipABC* operon, and *sseABCEFGJL* operon. There were some virulence genes present or absent in specific serotypes. For example, *faeE* gene, encoding periplasmic chaperone for K88 biosynthesis, was found only in serotype Anatum and Hvittingfoss, while *fepG* gene, encoding ferric enterobactin transport protein, was only detected in serotype Stanley. In contrast, *sifA* gene, encoding T3SS-2 effector protein, is present in all strains in this study except those of serotype Corvallis and Derby, while *avrA* gene, encoding an effector protein mediated host-immune response, is absent in serotype Amsterdam and Bovismorbificans.

According to *Salmonella* pathogenicity island (SPI) detection, eight SPIs, C63PI, and CS54 were detected (Table S7). All strains harbor SPI-1, SPI-2, SPI-3, and SPI-5. This finding was in concordance with virulence gene results which were T3SS genes located in SPI-1 and SPI-2, *mgtBC* or magnesium uptake gene in SPI-3, and *sopB* gene (phosphoinositide phosphatase gene, intracellular survival) and *pipB* (T3SS) in SPI-5. However, SPI-8 was limited to particular serotypes which were Krefeld, Kedougou, and Rissen. C63PI was found in most serotypes except serotype Amsterdam and Weltevreden. Conversely, only predicted serotype Tananarive or Brunei possessed CS54 island.

### Phenotypic AMR profiling

Based on antimicrobial susceptibility testing with 18 antibiotics, all strains were non-susceptible to at least one drug (Fig. 2). Surprisingly, 70% of isolates were MDR strains (not susceptible to ≥3 different drug classes). The majority of AMR patterns were ampicillin, streptomycin, and tetracycline resistance, accounting for 86.54% of the MDR isolates. According to tested drugs, our collection had a trend of streptomycin resistance (70/74, 94.59%) in concordance with the genotypic result which indicated that 73/74 strains carried an aminoglycosides resistance gene. The next most prevalent AMR patterns were ampicillin (51/74, 68.92%), ciprofloxacin (50/74, 67.57%), tetracycline (47/74, 63.51%), and amikacin (46/74, 62.16%). Moreover, there were seven isolates resistant to either cefotaxime or ceftazidime, and these strains were isolated from raw pork meat samples. Of these numbers, four strains were ESBL-producing strains, which were PP14-02 (*S*. Corvallis), PP14-08 (*S*. Derby), PP14-31 (*S*. Derby), and PP14-54 (*S*. Uganda). However, the most antibiotic-resistant strains were PP14-32 and PP14-35. Both strains were serotype Rissen, and they had the same resistance profile, which was resistance to a range of 13 to 18 drugs, but they were not ESBL-producing strains. None of the strains in this collection were carbapenem-resistant.

### Correlation between AMR genes or virulence genes and phenotypic antibacterial resistance profiles

Pearson correlation was used to analyze the correlation between phenotypic data from disk diffusion results and genotypic data from detected AMR and virulence genes. We assigned values for detected genes (genotype) as 0 = absent and 1 = present, while for phenotype, 0 = susceptible and 1 = intermediate resistance or resistant. If all isolates harbored particular genes or were susceptible to given drugs, the same calculated values were ignored. Based on all serotypes found in this study, we found a statistically significant and strong positive correlation between the presence of *tet(A*) gene and tetracycline resistance and ampicillin resistance phenotypes (Table S8). Presence of *floR* gene correlates with the ability to resist chloramphenicol. Gene *qnrS1* showed significantly positive correlation to ciprofloxacin resistance, while *aadA2*, *dfrA12*, *sul3*, *cmlA1*, and *qacE* positively correlated with sulfamethoxazole-trimethoprim resistance. The cluster of cefotaxime-ceftazidime-ceftriaxone-aztreonam resistance showed strong positive correlation together. The presence of *bla*_CTX-M-55_ positively correlated with the presence of aminoglycoside resistance genes (*aac (3)-IIc*, *aac (3)-Iie*, and *aadA22*) and norfloxacin resistance phenotype. The presence of *bla*_CTX-M-55_ also strongly positively correlated with the presence of *catA2* (chloramphenicol resistance). For the correlation between virulence genes and AMR phenotypes (Table S9), we found significant and strong positive correlation between *shdA* gene and norfloxacin resistance. Moreover, a negative correlation was revealed between *sseI*/*srfH* gene and streptomycin resistance. All findings described here were statistically significant with *P*-value < 0.01. Apart from the positive correlation of ESBL with third-generation cephalosporin group including monobactam resistance, no direct correlation for either AMR genes or virulence genes was found.

### Genomic characteristics of four *Salmonella* ESBL-producing strains

**Fig 4:**
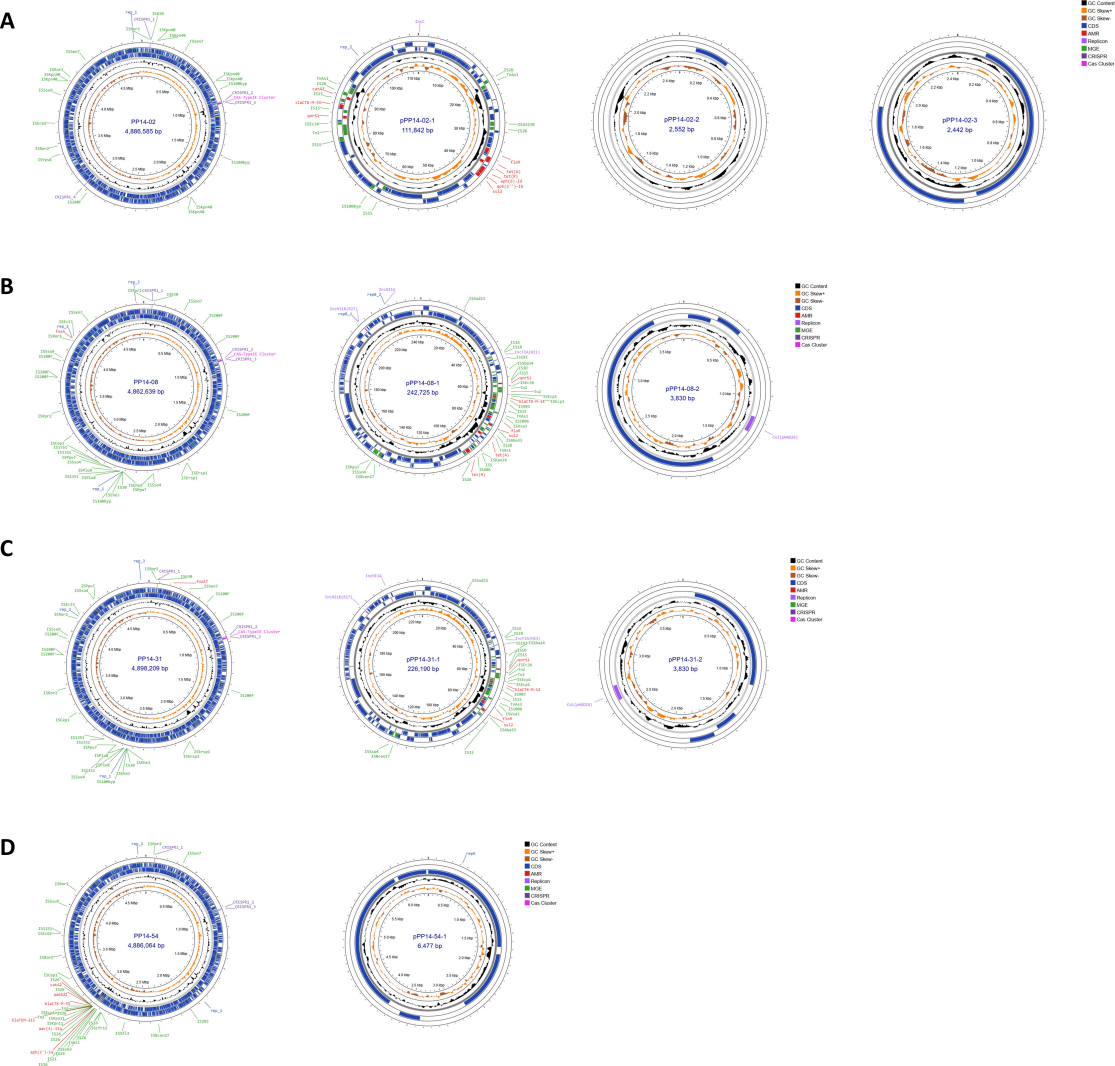
Complete genome visualization of ESBL-producing *Salmonella* strains. (**A**) Strain PP14-02. (**B**) Strain PP14-08. (**C**) Strain PP14-31. (**D**) Strain PP14-54.

Whole genome sequencing and hybrid assembly revealed the complete genome information of each ESBL-producing strain (Table S10; Fig. 4). Each strain contained one circular chromosome and at least one circular plasmid. According to annotation results, we found that most of the AMR genes were detected in plasmids except PP14-54 (Fig. 4D), in which all AMR genes were in the chromosome. All ESBL plasmids are larger than 100 kb in size with replicon and insertion sequences. Focusing on pPP14-02-1 (Fig. 4A), this plasmid had 92% similarity to pH1-012 from *Salmonella* serotype 4,[5],12:i:- strain H1-012 isolated from a Thai patient (Fig. S3). Both PP14-02 and H1-012 strains express resistance to and carry the *bla*_CTX-M-55_ gene and IncC plasmid, suggesting a wide distribution of this plasmid through the environment in Thailand. In the case of pPP14-08-1 and pPP14-31-1 (Fig. 4B and C), the *bla*_CTX-M-14_ gene is located in the IncFIA(HI1) plasmid and surrounded by many insertion sequences including other AMR genes, suggesting the mobility of this AMR plasmid. The isolation sources of *S*. Derby strains PP14-08 and PP14-31 are from different regions of Thailand, suggesting the common plasmids within the serotype.

### Intracellular infection efficiencies of four *Salmonella* ESBL-producing strains

**Fig 5:**
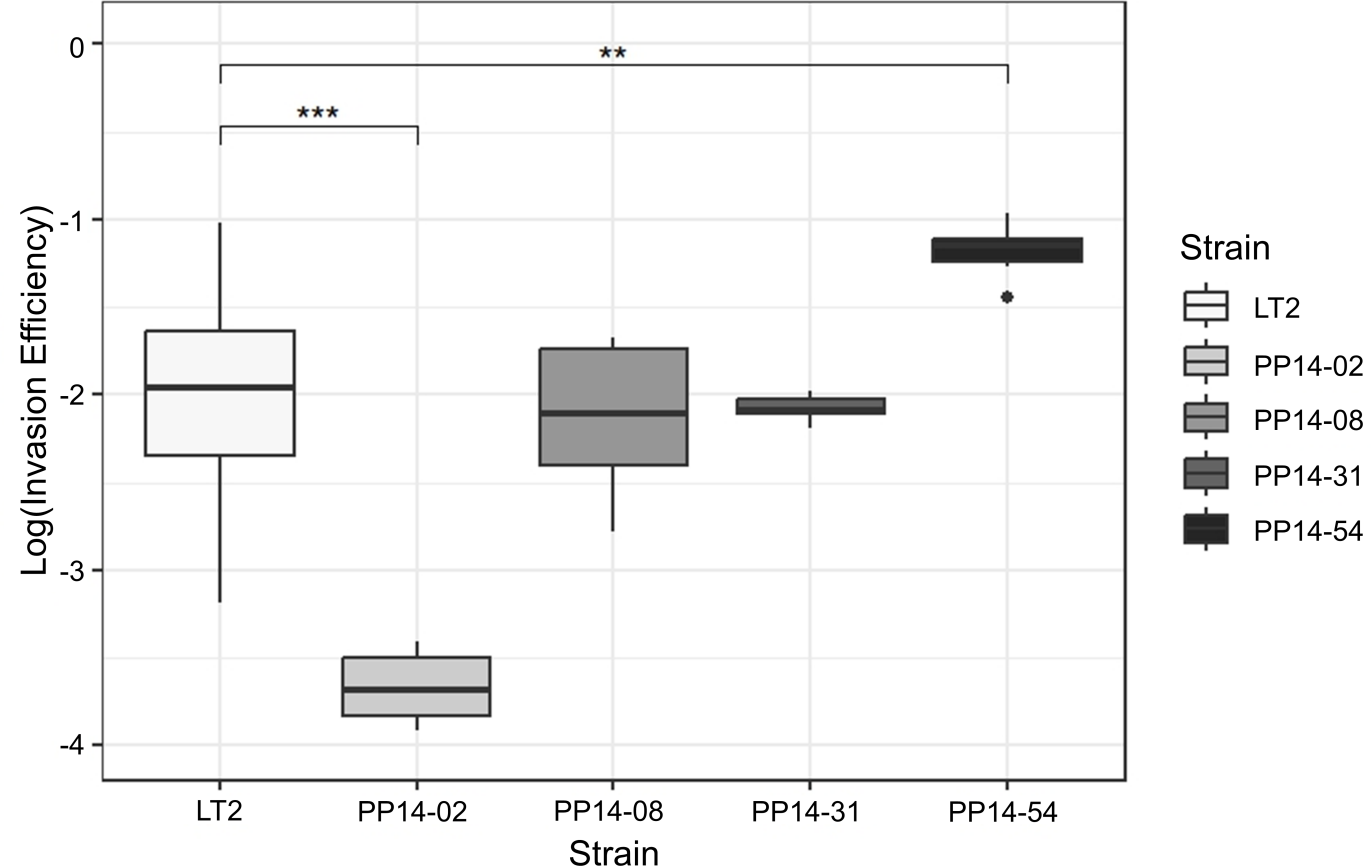
Invasion on HT29-MTX-E12 cells by four ESBL-producing *Salmonella* strains. Invasion efficiency (*X*-axis) is shown in the log difference between recovered bacteria and inoculum of the comparable strain (*Y*-axis). Experiments were performed in at least biological triplicates with technical duplicates. The statistically significant differences were determined based on one-way ANOVA with Dunnett’s post-hoc test to *Salmonella* Typhimurium LT2 and Bonferroni correction. The symbols *, **, and *** indicate the significant difference at *P* < 0.05, *P* < 0.01, and *P* < 0.001, respectively.

We further investigated intracellular invasion efficiency of four *Salmonella* ESBL-producing strains on HT29-MTX-E12 cells. The logarithm ratios of recovered bacteria to the inoculated bacteria were calculated, and *Salmonella* Typhimurium LT2 was used as a reference. The lower value indicated the higher invasion efficiency. As shown in Fig. 5, *Salmonella* strain PP14-54 had a higher ability to invade host cells than LT2 with *P* < 0.01, while *Salmonella* strain PP14-02 posed significantly lower efficiency with *P* < 0.001 compared to LT2. However, invasion efficiency values of PP14-08 and PP14-31 showed no statistical difference to LT2. PP14-54 has ESBL gene inside its chromosome region, while PP14-02 has three plasmids including ESBL harboring plasmid. The fitness cost of carrying plasmids may impact strain PP14-02 lowering its ability to invade human cells and undergo intracellular replication, especially in the absence of antibiotic.

## DISCUSSION

*Salmonella*-contaminated pork is one of the top sources of global food-borne salmonellosis (15). The increasing numbers of MDR *Salmonella* raise a public health concern, and they are now included in the 2024 Bacterial Priority Pathogens List issued by the WHO (6). *S*. Enteritidis and *S*. Typhimurium are the most common serotypes worldwide, but the prevalence of *Salmonella* serotypes can change based, in part, on differences in pathogenicity and virulence patterns. Thus, monitoring the changing prevalence and incidence of MDR *Salmonella* in food samples is necessary.

In this study, 74 *Salmonella* strains were isolated from 54 food samples (12). Based on whole genome sequencing, the most prevalent serotypes were, in descending order, Rissen ST469, Derby ST40, Stanley ST29, 4,[5],12:i:- ST34, and Kedougou ST1543. These serotypes are pathogenic to humans, especially in vulnerable groups, and were among the most common serotypes isolated from patients in Thailand during 2002–2007 (16). Serotype Rissen preferentially replicates intracellularly in human macrophages over murine macrophage-like cells. *S*. Rissen has also been frequently found in children with diarrhea in China (17, 18). Serotype Derby was responsible for a large outbreak in Germany that affected 145 primarily elderly people, with raw pork sausage identified as the probable source of infection (19). Serotype Stanley was the second most prevalent cause of human salmonellosis in Thailand in 2008. It is a known travel-associated pathogen that European patients acquired during travel to Southeast Asia (20). *S*. Stanley also caused a human outbreak from 2011 to 2013, resulting in 710 reported cases across 10 European Union countries (21). Another top pathogenic serotype is *S*. 4,[5],12:i:-, where the emergence of this monophasic serotype has been linked to human salmonellosis in various countries, including Thailand, since the 1990s (22, 23). Several human outbreaks have been traced back to contaminated pork meat and pork products (24, 25). Lastly, serotype Kedougou is commonly found in northern Thailand (26). In one notable case, a 64-year-old man developed travel-related bacterial enteritis from *S*. Kedougou infection after a 10-day stay in Chiang Mai, Thailand (27).

Surprisingly, none of the isolates included in this study were of serotype Enteritidis or Typhimurium, which usually are the most common serotypes worldwide (7). The predominant isolation of *S*. Rissen from pork retail meats in Thailand was in concordance with previous studies (9–11). Even though particular serotypes such as Agona, Give, or Corvallis were detected less frequently, they could still cause salmonellosis, and their MDR abilities could lead to treatment difficulty (28–32).

According to AMR gene prediction, all strains harbored at least one type of aminoglycoside-modifying enzymes. The *aac(6')-Iaa* gene, encoding aminoglycoside N-acetyltransferase (33), had the highest prevalence in our collection. This gene was suggested to be present in an ancestral strain and transmitted vertically through chromosome replication before serotype variation (34). Although *aadA* gene was found as the second most common aminoglycoside resistance gene in our collection, there was no statistically significant correlation between resistance genotype and phenotype.

The *bla*_TEM-1B_ gene showed the highest frequencies in β-lactamase group, and it is generally involved in resistance to first-generation cephalosporin such as ampicillin (35). The presence of the *bla*_TEM-1B_ gene was statistically significant and positively correlated to resistance to ampicillin with *P* < 0.01. Other β-lactamase genes that were detected in some strains were *bla*_CMY-2_, *bla*_CTX-M-14_, and *bla*_CTX-M-55_. Gene *bla*_CMY-2_ was usually found in plasmid-mediated β-lactamase (36). The *bla*_CMY-2_ plasmid can be transferred from *Escherichia coli* to *Klebsiella pneumoniae* (37), suggesting the transfer may occur among *Enterobacteriaceae* within the same environment including the same food sample. In our study, the *bla*_CMY-2_-positive strains were all isolated from raw meat from Kanchanaburi province, which might imply the spread of specific β-lactamase gene in the western part of Thailand. Correlation analysis revealed the positive correlation with statistical significance between the presence of *bla*_CMY-2_ gene and amoxicillin/clavulanate resistance. The two *S*. Derby isolates carried *bla*_CTX-M-14_ in their plasmids. Though many studies reported discovering *bla*_CTX-M-14_ in *E. coli* and *Salmonella* Typhimurium (38), no previous studies have mentioned *Salmonella* Derby carrying this type of β-lactamase gene. Gene *bla*_CTX-M-55_ is one of the common CTX-M ESBL subtypes first discovered in Thailand (39). Recently, *bla*_CTX-M-55_ was still detected in *Salmonella* isolated from canals or produce commodities in Bangkok, Thailand (40, 41), whereas our study strains (*S*. Corvallis PP14-02 and *S*. Uganda PP14-54) were isolated from raw pork meat sold in fresh markets in Chiang Rai (Northern region) and Prachuap Khiri Khan (Southern region), respectively. Together with the complete genome results, *bla*_CTX-M-55_ gene of both strains was in an AMR region flanked with IS*26*, an insertion sequence that plays a major role in genetic rearrangement, plasmid reorganization, and cointegrates formation (14, 42). In summary, the circulation of the *bla*_CTX-M-55_ gene, including the *Salmonella* carrying β-lactamase genes in the environment and food products, is high throughout Thailand.

Based on virulence gene prediction, all *Salmonella* strains in this study harbored many genes involved in *Salmonella* persistence in the environment such as adherence, chemotaxis, siderophores, and T3SS. Presence of these genes suggested the potential of strains from food samples to colonize and infect humans (43–46). Our study revealed that some of the virulence genes were present or absent in specific serotypes. For example, the *faeE* gene was detected only in serotype Anatum and Hvittingfoss, while the *fepG* gene was limited to serotype Stanley. The *lpfABCDE* operon was absent in serotype Amsterdam, Anatum, Corvallis, Derby, Give, Uganda, and Weltevreden. This virulence operon encodes for long polar fimbriae and is involved in attachment to murine Peyer’s patches (47, 48). Further investigation is needed to confirm these strict correlations. All *Salmonella* strains contained SPI-1, SPI-2, SPI-3, and SPI-5, indicating the necessity of these SPIs for *Salmonella* persistence in the environment (49, 50). SPI-8 had been initially reported to be specific to serotype Typhi and Paratyphi A but was later found in other serotypes such as Enteritidis, Washington (51, 52). In this study, we identified the presence of SPI-8 in serotypes Kedougou, Krefeld, and Rissen, supporting that *Salmonella* isolated from food in Thailand harbors not only essential genes for their persistence in the environment but also virulence genes for invasion into human cells along with enhanced survival in host cell.

Antibiotic susceptibility testing on the isolates raised significant public health concerns. First, 70% of our isolates were MDR strains with the majority of AMR patterns resistant to ampicillin, streptomycin, and tetracycline. Second, more than 50% were intermediately resistant to ciprofloxacin. Resistance to quinolone class drugs negatively affects systemic salmonellosis treatment, resulting in treatment failure. Lastly, four ESBL-producing strains consisting of *S*. Corvallis, *S*. Derby, and *S*. Uganda were detected from raw pork, and their origins were from different regions in Thailand. *S*. Rissen strains with resistance to third-generation cephalosporin and monobactam, but not considered as ESBL producers, were detected.

The invasion assay showed insight on how virulent each isolate was on the intestinal human cell line infection. An ESBL-producing strain carrying AMR genes including the ESBL gene on its chromosome had a higher invasion efficiency compared to the reference strain *S*. Typhimurium LT2. Plasmid-mediated ESBL strains have less invasion abilities, and this could be due to the fitness cost of maintaining plasmids. Even though plasmid-mediated ESBL strains caused less virulence than the chromosomal ESBL strain, they could still cause a challenge in treatment, especially in immunocompromised patients (53).

The emergence of MDR *Salmonella* in Thailand poses serious threats to public health, particularly for vulnerable groups who are at a higher risk of invasive diseases and complications (3, 54). This emergence is associated with prolonged hospital stays, increased treatment costs, and higher morbidity due to limited therapeutic options (55). One of the most important factors driving the emergence of MDR bacteria is the misuse of antibiotics in both animals and humans. This practice creates environmental pressure that selects for antibiotic-resistant strains (56, 57). To address this, Thailand introduced the Antibiotics Smart Use program in 2007 to promote the rational use of antibiotics and discourage their use for non-bacterial infections (58). However, significant challenges remain. Over-the-counter antibiotics are still easily accessible without a prescription, and there is a lack of the resources and capacity to audit antibiotic prescriptions effectively (54, 58). A strong political commitment to combat AMR is needed in order to successfully decrease antibiotic misuse by health professionals and to ensure all healthcare units follow the ASU treatment guidelines (58).

Overall, our study found that AMR genes in the plasmid-mediated ESBL strains were flanked with insertion sequences indicating the possibility of transferring an MDR plasmid among bacteria in the same food environment via horizontal gene transfer. As a country in an economic region that relies heavily on food production, food export, and tourism, this report aims to raise the awareness of limited antibiotic use in food animals and remediation of drug spillovers in the environment and landfills.

## MATERIALS AND METHODS

### *Salmonella* isolates from food samples

Seventy-four *Salmonella* isolates collected from March to April 2018 (12) were used in this study. Isolates were retrieved from −80°C storage and cultured in Tryptic Soy Broth (TSB) (BD, USA) at 37°C overnight prior to experiments.

### Genomic DNA extraction and short read sequencing

Bacterial genomic DNA was extracted from each 5 mL overnight *Salmonella* culture in TSB by using QIAamp DNA Mini Kit (QIAGEN, Germany). After proteinase K lysis step, a total of 0.2 mg RNase A (Vivantis, Malaysia) was added and incubated samples were incubated at 37°C for 1 h as an additional step before following the kit protocol. Nuclease-free water or 10 mM Tris-Cl pH 8.0 was used to elute DNA. The quantity of extracted genomic DNA (gDNA) was measured by NanoDrop ND-1000 Spectrophotometer (Thermo Fisher Scientific, USA) and Qubit dsDNA BR assay kit (Invitrogen, USA) while the quality was determined by 1% agarose gel electrophoresis in 1× Tris-acetate-EDTA buffer.

Sixty purified extracted gDNAs were sent to the Division of Infectious Diseases Wadsworth Center, New York State Department of Health, NY (NYSDOH) for short read whole genome sequencing using Illumina NextSeq500 instrument. Next-generation sequencing libraries were prepared in the Wadsworth Center Applied Genomic Technologies Cluster with the Illumina DNA Prep kit, using a modified, quarter scale protocol (59). Other purified samples were sent to BGI Hong Kong for sequencing using an MGI DNBSEQ sequencer and using MGIEasy FS DNA Library Prep Set (MGI, China) for library preparation.

### Genome assembly and analysis

Raw reads were assessed for their quality by using FastQC v0.12.0 (60). The reads were trimmed using Trim Galore v0.6.5dev (61) and *de novo* assembled using SPAdes genome assembler v4.0.0 (62) via BV-BRC web resource (63). The assembled contigs were assessed for their quality, contiguity, and correctness in comparison to the complete genome of *Salmonella* Typhimurium LT2 (accession number: NC_003197.2) via the Galaxy website (64) using QUAST v5.3.0 (65). Completeness and contamination were assessed on the Galaxy platform using BUSCO v5.5.0 (66) run locally against enterobacterales_odb10 database and checkm2 v1.0.2 (67).

ST and clonal complex were determined based on seven loci MLST (*aroC*, *dnaN*, *hemD*, *hisD*, *purE*, *sucA*, and *thrA* genes), and PubMLST (68). SeqSero2 was used for serotyping according to the Kauffmann-White scheme (69). Assembled contigs were annotated using the RAST tool kit (RASTtk) by BV-BRC version 3.30.19 (63). A phylogenetic tree was constructed based on Codon Tree method in BV-BRC using *Salmonella enterica* subsp. *indica* (NCBI Sequence Read Archive: SRR2585491) as an outgroup and visualized by Interactive Tree Of Life (iTOL) version 6 (70). Antibiotic resistance genes were predicted by the Comprehensive Antibiotic Resistance Database (71), the National Database of Antibiotic Resistant Organisms (72), and ResFinder v4.6.0 (73, 74). PlasmidFinder 2.1 v2.0.1 was used for typing and identifying plasmid replicons (75), while MobileElementFinder v1.0.3 was used to detect MGEs (76). SPIs were detected using SPIFinder 2.0 (77). VFDB was used to predict virulence factor genes (78). Predicted antibiotic resistance or virulence genes and detected MGEs were called if both identity and gene coverage percentages were >90% and *E*-values were less than 0.01.

### Antimicrobial susceptibility testing

Antimicrobial susceptibility testing was determined by disk diffusion method following Clinical and Laboratory Standard Institute (CLSI) guideline (79). In this study, 74 NTS isolates were tested on 18 antibiotics (amikacin [AN, 30 µg, BD BBL, USA], amoxicillin/clavulanic acid [AMC, 20/10 µg, HiMedia, India], ampicillin [AM, 10 µg, BD BBL, USA], aztreonam [ATM, 30 µg, BD BBL, USA], cefotaxime [CTX, 30 µg, BD BBL, USA], ceftazidime [CAZ, 30 µg, BD BBL, USA], ceftriazone [CRO, 30 µg, BD BBL, USA], chloramphenicol [C, 30 µg, BD BBL, USA], ciprofloxacin [CIP, 5 µg, BD BBL, USA], gentamicin [GM, 10 µg, BD BBL, USA], imipenem [IPM, 10 µg, BD BBL, USA], kanamycin [K, 30 µg, Oxoid, UK], meropenem [MEM, 10 µg, BD BBL, USA], nalidixic acid [NA, 30 µg, Oxoid, UK], norfloxacin [NOR, 10 µg, BD BBL, USA], streptomycin [S, 10 µg, Oxoid, UK], tetracycline [Te, 30 µg, BD BBL, USA], and sulfamethoxazole-trimethoprim [SXT, 23.75/1.25 µg, BD BBL, USA]). Inhibition zone was measured and interpreted following CLSI (Table S12). CTX or CAZ non-susceptible strains (inhibition zone less than 26 and 21 mm, respectively) were further tested for ESBL production by phenotypic confirmatory disk diffusion test (PCDDT) (79). Single antibiotic disks (CAZ [30 µg] and CTX [30 µg], BD BBL, USA) were used to compare with those combined with clavulanic acid (CLA) i.e., CAZ/CLA (30/10 µg) and CTX/CLA (30/10 µg) (BD BBL, USA). If the inhibition zone of combined antibiotic disk was larger than single antibiotic disk by 5 mm, it was confirmed that the tested strain was ESBL-producing. The experiment was performed in biological triplicates, and *E. coli* ATCC 25922 was used as a quality control strain.

### Correlation and statistical analysis

Pearson’s correlation coefficient was used to analyze the correlation between AMR genotype and phenotype (80). The correlation coefficient values were interpreted as follows: 0.90 to 1.00 as very strong positive correlation, 0.70 to 0.90 as strong positive correlation, −0.70 to −0.90 as strong negative correlation, and −0.90 to −1.00 as very strong negative correlation. The correlation between two groups was considered statistically significant when the *P*-value was less than 0.05. However, IPM and MEM resistance phenotypes were rejected from correlation analysis since all strains were sensitive to these drugs.

### Long-read sequencing and hybrid genome assembly

Four ESBL-producing strains were cultured and gDNA samples were extracted. After passing quality and quantity measurements described above, the DNA Integrity Number was used to determine the quality of extracted gDNA using Genomic DNA ScreenTape assay with 4200 TapeStation System (Agilent, USA). A total of 1 µg purified gDNA was used as input for library preparation using Ligation sequencing gDNA-Native Barcoding Kit 24 V14 (SQK-NBD114.24). DNA repair and end-prep steps were performed as described for NEBNext FFPE DNA Repair Mix and NEBNext Ultra II End Repair/dA-Tailing (New England Biolabs, USA), respectively. AMPure XP Beads (AXP, 0.8 vol) were used to clean up after native barcode ligation step. In the native adapter ligation step, Blunt/TA Ligase Master Mix (New England Biolabs, USA) was used prior to 0.8 vol AXP cleanup. Prepared library was loaded into MinION flow cell R10.4.1 (Oxford Nanopore Technologies, UK) and sequenced using Mk1C sequencer (Oxford Nanopore Technologies, UK).

Resulting raw long reads were concatenated into one fastq file on Galaxy (81), and the mean read quality was assessed using Nanoplot v1.44.1 (82). Adapters were trimmed using Porechop v0.2.4, and all trimmed reads were filtered with the minimum length of 1,000 bp using Filtlong v0.2.1 (83). Filtered reads were hybrid assembled with trimmed short read sequences using Unicycler v0.5.1 (84). All complete genomes were annotated using NCBI Prokaryotic Genome Annotation Pipeline and visualized using Proksee (85).

### Invasion assay

The mucus-producing intestinal cell line HT29-MTX-E12 was used in this study. HT29-MTX-E12 cells were cultured and routinely maintained in T-75 flasks at 37°C and 5% CO_2_ in complete Dulbecco’s Modified Eagle’s medium (DMEM) (Gibco, USA) containing 10% fetal bovine serum (Gibco, USA), 1 × non-essential amino acids (Gibco, USA), and 1 × L-glutamine (GlutaMAX, Gibco, USA). The culture media were changed every 72 h. For *Salmonella* invasion assay, cells were seeded into 24-well plates approximately 1.5 × 10^5^ cells and grown for 5 days prior to infection. Two representative wells of HT29-MTX-E12 cells were washed with phosphate-buffered saline (PBS) and dissociated with 0.25% Trypsin-EDTA (Gibco, USA) in order to determine the number of cells. Overnight cultures of ESBL-producing *Salmonella* strains were diluted 1:30 in 5 mL of Luria-Bertani (LB) broth and grown at 37°C, 200 rpm until OD600 was in the range 0.6–0.8. One milliliter of bacterial cells was pelleted, resuspended in 1 mL of PBS, and diluted in DMEM with the ratio for infection at a multiplicity of infection of 15 (15 bacterial cells: 1 host cell). Two representative wells of HT29-MTX-E12 cells were washed with PBS before addition of bacterial cells. After 60-min incubation at 37°C and 5% CO_2_, infected HT29-MTX-E12 cells were washed three times with PBS before addition of 300 µg/mL gentamicin in DMEM and incubated for 60 min in order to kill remaining extracellular bacterial cells. The cells were washed three times with PBS and lysed with 0.1% sodium dodecyl sulfate. The numbers of intracellular bacterial cells and inoculum were determined by viable cell count method using LB agar plate. Invasion efficiency was calculated as log10 (number of recovery bacteria/number of bacterial inoculum). The experiment was performed in biological triplicates, and *Salmonella* Typhimurium LT2 was used as a reference strain. For comparison, one-way analysis of variance (ANOVA), Dunnett’s post-hoc test, and Bonferroni correction were used, and *P* < 0.05 was considered as statistically significant difference.

## Data Availability

The short-read sequencing data have been deposited under BioProject accession number PRJNA183850 and PRJNA1227446. The long-read sequencing data and complete genomes are available under BioProject accession number PRJNA1182244.

## SUPPLEMENTAL MATERIAL

Supplemental figures spectrum.02129-25-s0001.docx Fig. S1 to S3.

Supplemental tables spectrum.02129-25-s0002.xlsx Tables S1 to S12.

ASM does not own the copyrights to Supplemental Material that may be linked to, or accessed through, an article. The authors have granted ASM a non-exclusive, world-wide license to publish the Supplemental Material files. Please contact the corresponding author directly for reuse.
