# Supplementary material for: High prevalence of multidrug-resistant Salmonella enterica in Thailand food markets: insights from complete genome and phenotypic characterization of ESBL-producing strains
Source: Microbiol Spectr. 2025 Oct 31;13(12):e02129-25. doi: 10.1128/spectrum.02129-25 (PMC12671207; doi:10.1128/spectrum.02129-25)
Supplement: Supplemental figures — Fig. S1 to S3. [file spectrum.02129-25-s0001.docx]

**Supplementary figures**

**High prevalence of multidrug resistant *Salmonella enterica* in Thailand food markets: insights from complete genome and phenotypic characterization of extended-spectrum β-lactamase (ESBL)-producing strains**

**Authors:** Phutthaphorn Phaophu^1^, Nalumon Thadtapong^2^, Samantha E. Wirth^3^, Anna S. Gray^3^, Sunisa Dangsuk^1^, Natharin Ngamwongsatit^4^, Ratchaneewan Aunpad^5^, Soraya Chaturongakul^1,6#^

^1^Center for Advanced Therapeutics, Institute of Molecular Biosciences, Mahidol University, Nakhon Pathom 73170 Thailand

^2^National Institute of Health, Department of Medical Sciences, Ministry of Public Health, Nonthaburi, 11000 Thailand

^3^New York State Department of Health, Wadsworth Center, Albany, New York, 12208 USA

^4^Department of Clinical Sciences and Public Health, Faculty of Veterinary Science, Mahidol University, Nakhon Pathom 73170 Thailand

^5^Graduate Program in Biomedical Sciences, Faculty of Allied Health Sciences, Thammasat University, Pathum Thani, 12121 Thailand

^6^Pornchai Matangkasombut Center for Microbial Genomics (CENMIG), Faculty of Science, Mahidol University, Bangkok 10400 Thailand

^#^Address correspondence to Soraya Chaturongakul, soraya.cha@mahidol.ac.th.


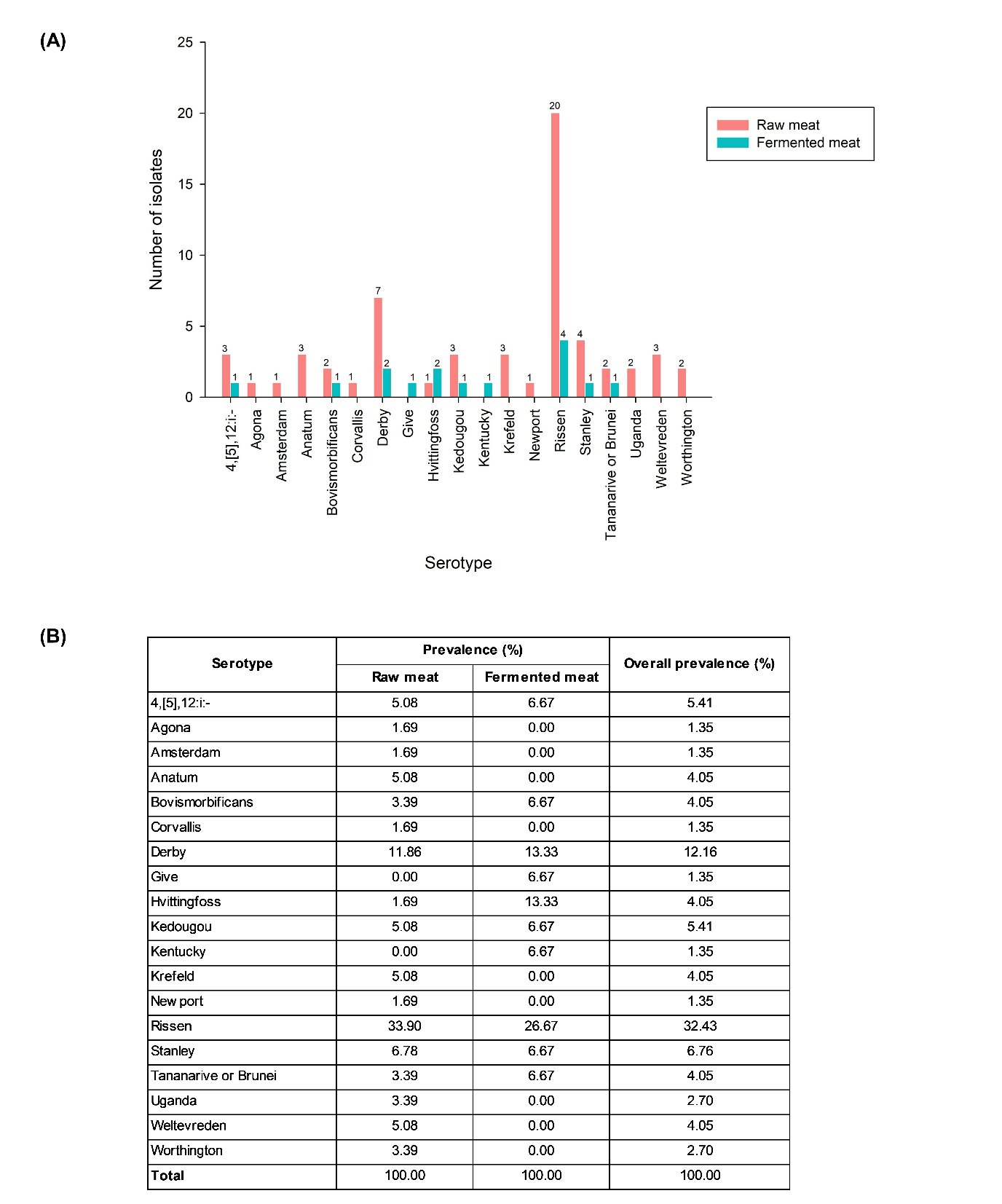


**Fig. S1.** The prevalence of *Salmonella* serotypes isolated from food samples. (A) Histogram of serotype prevalence in different food samples. Pink indicates raw meat while green indicates fermented meat. (B) Percent prevalence of *Salmonella* serotypes in raw meat and fermented meat. Overall percent prevalence calculated by the total number of particular serotype divided by total isolate numbers (74 isolates).


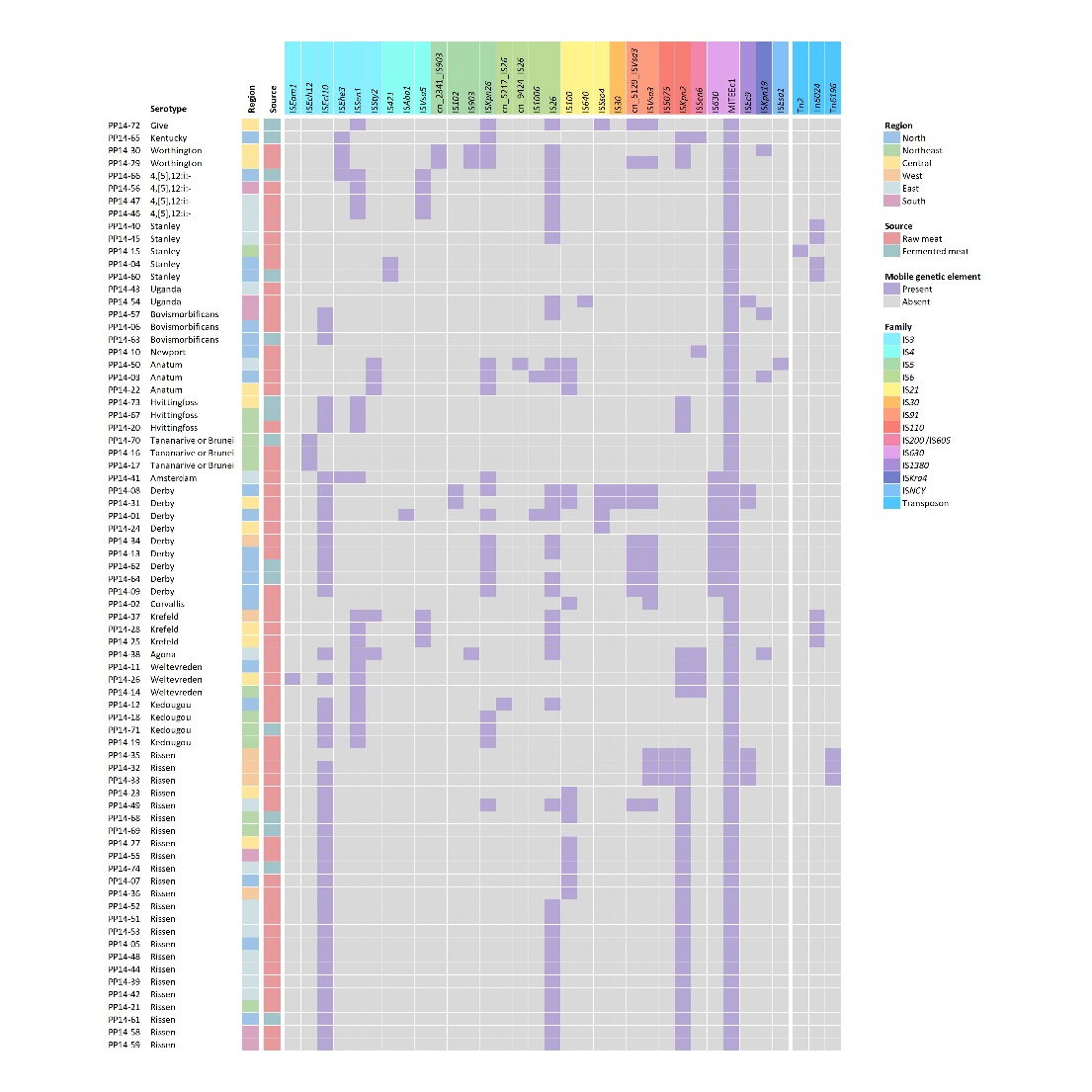


**Fig. S2.** Heatmap of mobile genetic elements detected in *Salmonella* isolates. Presence and absence are respectively shown in purple and gray. Different colors in mobile genetic elements represent different mobile genetic element family which are labeled next to the heatmap.


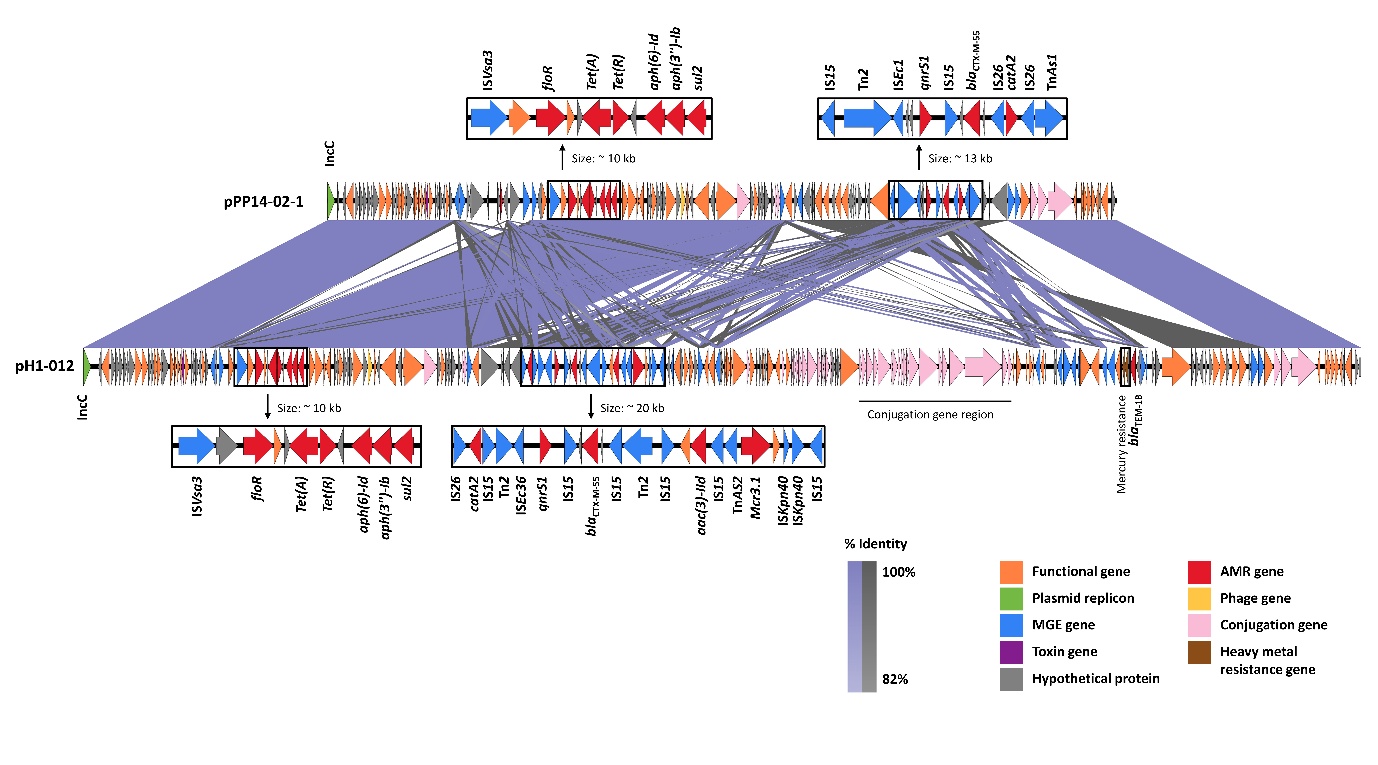


**Fig. S3.** Comparative gene maps of plasmid pPP14-02-1 and pH1-012. Both carry antimicrobial resistance gene clusters including *bla*_CTX-M-55_ gene and IncC plasmid.
